# The Thin Ideal and Attitudes towards Appearance as Correlates of Exercise Addiction among Sporty People during the COVID-19 Pandemic

**DOI:** 10.3390/bs12060187

**Published:** 2022-06-11

**Authors:** Rubinia Celeste Bonfanti, Gianluca Lo Coco, Laura Salerno, Maria Di Blasi

**Affiliations:** Department of Psychology, Educational Science and Human Movement, University of Palermo, 90128 Palermo, Italy; gianluca.lococo@unipa.it (G.L.C.); laura.salerno@unipa.it (L.S.)

**Keywords:** COVID-19, exercise addiction, moderate-to-vigorous physical activities, drive for leanness, sociocultural attitudes towards appearance

## Abstract

The stress and anxiety caused by the coronavirus pandemic (COVID-19) have exacerbated body image concerns. A society that perpetuates the attempt for a perfect and thin appearance represents a fertile ground for the development of exercise addiction (EA). This cross-sectional study aims to explore EA during the second wave of the pandemic (October–December 2020) and to examine the independent influence of both time spent on moderate and vigorous physical activities and body image variables (i.e., drive for leanness and sociocultural attitudes toward appearance) on EA. A sample of Italian sporty people (N = 194; 48.5% females; M_age_ = 25.91 ± 6.32) was surveyed using the Exercise Addiction Inventory, the Drive for Leanness Scale, the Sociocultural Attitudes Towards Appearance Questionnaire, and the Global Physical Activity Questionnaire. A total of 82% of the sample were symptomatic of and 11.3% were at risk of EA. Hierarchical regressions revealed an association between the time spent on vigorous physical activities and levels of EA (*p* < 0.05). Moreover, body image variables were positively related to EA, explaining an additional 11% of variance (*p* < 0.05). Results showed the importance of considering and addressing body image factors to investigate and dampen the risk of EA among sporty people.

## 1. Introduction

The coronavirus disease (COVID-19) pandemic has brought dramatic changes to everyday life [1,2]. Scholars argued that the experience of lockdown and home confinement due to the pandemic has been an undesirable condition, which has negatively impacted individuals’ wellbeing [3,4]. Since the COVID-19 outbreak, there is numerous evidence showing high rates of post-traumatic stress disorder in the general population, as well as an increase in symptoms of isolation, confusion, anger, frustration, anxiety, and depression [5,6,7]. Among the different areas of daily life, sports activities were also affected by lockdown and other common strategies to mitigate the effects of the pandemic, leading to various levels of physical inactivity. The abrupt closure of gyms and fitness centers, the deferring of sporting events, as well as restrictions in outdoor activities have led to physical, psychological, and behavioral consequences for people who regularly exercise [8].

It is well known that habitual physical activity has physical and mental health advantages [9,10]. Frequently, people are involved in a physically active lifestyle for health reasons, such as coping with stress [11,12]. Despite their coping function, moderate levels of exercise and sports activities yield a beneficial effect on mental health [13]. However, prior research suggested that a physically active lifestyle could become compulsive [14] and may urge individuals to progressively increase their own exercise level to attain more results [15]. Although the possible negative effects of over-exercising on an individual’s well-being, to date, this kind of repetitive and problematic physical activity has not received a formal recognition as a mental disorder and has not yet been included in DSM-5 [16]. In this study, the construct of exercise addiction (EA) will be used to describe a morbid pattern of behaviors in which an individual loses control over his/her exercise habits, by showing the six components of behavioral addictions described by Griffiths [17] (i.e., salience, mood modification, tolerance, withdrawal symptoms, conflict, and relapse). Among sporty people, the risk of developing EA has been estimated to be close to 10% [18]. Previews studies describe EA related to certain factors, such as reward processes, repetitiveness, mood change, stress reduction, and anxiety alleviation [19,20]. However, the characteristics of excessive exercising or EA among exercisers during the COVID-19-related restrictions have received limited research attention [21]. Some preliminary findings showed that, although the decrease in exercise volume was common, the risk for EA or problematic exercising was high [15,22].

Increased anxiety and stress caused by the pandemic may represent a threat to body image for sporty people [23]. In this regard, numerous media can influence sociocultural attitudes towards appearance promoting an ultra-thin ideal contributing to an internalization of a skewed body image and problematic eating behaviors, especially during the pandemic [24]. This occurs because COVID-19-related pressures and stress may decrease individual coping resources to control threats related to body image, and may increase the need to exhibit idealized thin and athletic bodies through social media photos [8,25]. Sociocultural attitudes towards appearance consist of a disproportional emphasis of an ultra-fit, toned body as the cultural ideal, with a consequent internalization of these ideals [26]. Internalization is linked to a cognitive approval of the cultural ideal of an agreeable appearance. It includes asserting the attractiveness of socially defined ideals and drawing in specific behaviors to attain those ideals [27]. For this reason, internalization of appearance ideals is now regarded as a risk factor [28], especially for sporty people [29]. Some research has highlighted that the internalization of appearance ideals is a predictor of EA at both cross-sectional and longitudinal levels [26,30,31]. Moreover, prior studies have also shown significant associations between a drive for leanness and EA [32]. Drive for leanness refers to the tendency to extreme aspiration of being thin and being afraid of gaining weight, as well as a particular recognition of diet concerns [33]. It has frequently been found to be a significant predictor of compulsive exercise in cases where losing weight is one of the main personal goals [34,35,36].

To date, few preliminary studies show the antecedents of compulsive exercise under normal conditions [29], but there is a dearth of research on the role of the correlates of EA during the pandemic. To our knowledge, no previous studies have examined the relationship between physical activity level (i.e., time spent on moderate and vigorous physical activity), drive for leanness, sociocultural attitudes towards appearance, and EA during the prolonged pandemic restrictions due to the COVID-19 outbreak [37].

The general aim of the current study is to examine the link between EA and body image concerns among exercisers during the pandemic. The specific aims are twofold: (1) to explore EA in a sample of Italian sporty people who usually attended the gym during the second wave of the COVID-19 pandemic, and (2) to examine the independent contribution of both physical activity level (i.e., time spent on moderate and vigorous physical activity) and body image variables (i.e., drive for leanness and sociocultural attitudes towards appearance) to EA. It was hypothesized that body image variables (i.e., drive for leanness and sociocultural attitudes toward appearance) would explain additional variance in EA over and above the physical activity level. More specifically, higher levels of drive for leanness and sociocultural attitudes towards appearance would be related to higher EAs.

## 2. Materials and Methods

### 2.1. Participants

One hundred and ninety-seven people were recruited through convenience sampling and agreed to complete an online survey. Three subjects were subsequently excluded because of missing data on key variables. Thus, the final sample was composed of 194 amateur sporty people with regular membership at a CrossFit gym. The participants’ demographics and health-related information are reported in Table 1.

### 2.2. Measures

The first part of the questionnaire was used to collect information about the participants’ characteristics, including age, gender, level of education, marital status, current gym membership, current height, and the last weight taken with the weighing machine at the gym (body mass index—BMI—was subsequently derived from self-reported data for current height and weight; weight/height^2^). In the second part, data about time spent on moderate-to-vigorous physical activity, EA, drive for leanness, and sociocultural attitudes towards appearance were collected.

#### 2.2.1. Time Spent on Moderate and Vigorous Physical Activity (MVPA)

Self-reported weekly physical activity was assessed using the recreational activities domain of the *Global Physical Activity Questionnaire* (GPAQ) developed by the WHO [38]. The recreational activities subscale from the GPAQ comprises 6 questions, 3 referred to vigorous activity (e.g., *“In a typical week, on how many days do you do vigorous-intensity sports, fitness or recreational (leisure) activities?”*) and 3 referred to moderate activity (e.g., *“In a typical week, on how many days do you do moderate-intensity sports, fitness or recreational (leisure) activities?”*), with different response options for each item. The GPAQ estimates the minutes spent on moderate- and vigorous-intensity physical activity in a typical week.

#### 2.2.2. Exercise Addiction (EA)

EA was assessed with the *Exercise Addiction Inventory* (EAI) [39]. The EAI is a 6-item unidimensional measure (e.g., “*Exercise is the most important thing in my life*”). Items were rated on a 5-point Likert scale, from 1 (*Strongly Disagree*) to 5 (*Strongly Agree*), with higher overall scores indicating a higher risk of EA. A score of 0–12 indicates asymptomatic individuals, a score of 13–23 is indicative of symptomatic individuals, and a cut-off score of 24 indicates subjects at risk of EA [17,39]. The EAI showed an acceptable internal reliability in the present study (Cronbach’s α = 0.62; mean inter-item correlation = 0.213).

#### 2.2.3. Drive for Leanness (DL)

Drive for leanness was assessed with the *Drive for Leanness Scale* (DLS) [40]. DLS is a 6-item unidimensional measure (e.g., “*Athletic looking people are the most attractive people*”). Items were rated on a 6-point Likert scale, from 1 (*Never*) to 6 (*Always*), with higher overall scores indicating greater attitudes towards a lean and well-toned body. The DLS showed good internal reliability in the present study (Cronbach’s α = 0.85).

#### 2.2.4. Sociocultural Attitudes towards Appearance

Sociocultural attitudes towards appearance were assessed with the *Sociocultural Attitudes Towards Appearance Questionnaire-4* (SATAQ-4) [41]. The SATAQ-4 is a 10-item measure (e.g., *“I want my body to look very thin”*). Items were rated on a 5-point Likert scale, from 1 (*Definitely Disagree*) to 5 (*Definitely Agree*), with higher overall scores indicating a greater internalization. The SATAQ-4 showed good internal reliability in the present study (Cronbach’s α = 0.84).

### 2.3. Procedure

Respondents for this study were recruited both online and in-person through advertisements that were posted on social media sport forums and Italian gyms’ websites. The recruitment announcement included a link to the survey. The online survey took place between 30 October 2020 and 30 December 2020 (during the second wave of the COVID-19 pandemic in Italy). The questionnaire was anonymous, but the respondents could indicate their willingness to know the results and provide their email details. The online questionnaire took approximately 10–15 min to be completed. The research was conducted in accordance with the ethical standards of the Italian Psychological Association (AIP) as well as the Declaration of Helsinki. All participants completed statements of informed consent to participate in the study.

### 2.4. Statistical Analysis

A preliminary analysis was conducted to verify the normality of data distribution (i.e., skewness and kurtosis). A positive skewed distribution for time spent on vigorous physical activity was found and a square root transformation was conducted to normalize these data. All other variables revealed no substantial violation of normality regarding data distribution (|Sk| < 1; |Ku| < 1; see Table 2). In order to determine the internal consistency of all scales, Cronbach α and mean inter-item correlations were computed. Mean inter-item correlations between 0.15 and 0.50 indicate adequate internal consistency [42]. Descriptive statistics (mean and standard deviations for continuous variables and frequencies and percentages for categorical variables) and bivariate correlation coefficients (Pearson’s correlation coefficients) between variables were examined. A three-step hierarchical regression analysis was performed to verify the direct effect of time spent on moderate- and vigorous-intensity physical activity and body image variables (i.e., drive for leanness and sociocultural attitudes towards appearance) on EA. Control variables (i.e., age and gender) were entered into the model in the first step. Time spent on moderate- and vigorous-intensity physical activity was entered in the second step. Finally, body image variables (i.e., drive for leanness and sociocultural attitudes towards appearance) were entered in the third step. Analyses were performed using IBM SPSS Statistics (Version 26). The level of significance chosen for all analyses was 0.05.

## 3. Results

### 3.1. EA in Italian Sporty People during the Second Wave of the COVID-19 Pandemic

The number of male and female participants was 94 and 100, respectively. As shown in Table 2, an average score of 18.49 (±3.99) was reported in the Exercise Addiction Inventory. The vast majority of the samples (n = 159, 82.0%) are symptomatic for EA, whereas twenty-two (11.3%) participants are at risk of EA (mostly males; 68.2%). Only thirteen participants (6.7%) reported no EA symptoms. Table 2 shows the descriptive values (means and standard deviations) and correlations for the study variables. Pearson’s product-moment correlations indicated that EA is positively and significantly associated with sociocultural attitudes towards appearance, DL, and time spent on MVPA.

### 3.2. Univariate Analysis of the Association between Time Spent on MVPA, Body Image Variables, and EA

Regression analysis (Table 3) showed that the first model was not significant, and the two control variables were not significantly associated with EA. The second model showed the effect of time spent on vigorous physical activity in predicting EA. The results reveal that changing the model is significant (*p* < 0.01) and that the introduction of time spent on moderate and vigorous activity explains a 5% additional variance in EA. More specifically, when the time spent on vigorous physical activity increases by 1 point, the EA increases by 0.163 points. The effect of the control variables remained unchanged when compared to the previous model. The addition of the body image variables (i.e., sociocultural attitudes towards appearance and DL) in the third model explained an additional 11% of variance. Higher levels of both sociocultural attitudes towards appearance and DL were related to higher EA. The effect of the control variables, as well as the time spent on moderate and vigorous physical activity, remained unchanged when compared to the previous model. More specifically, when the time spent on vigorous physical activity increased by 1 point, the EA increased by 0.179 points; when the sociocultural attitudes toward appearance increased by 1 point, the EA increased by 0.166 points; and, finally, when the DL increased by 1 point, the EA increased by 0.215 points.

## 4. Discussion

Although moderate exercise has numerous health benefits, some individuals may become excessively preoccupied with exercise, endorsing addiction features. The current study examined the prevalence of EA in a sample of Italian amateur sporty adults during the COVID-19 pandemic, as well as the effect of time spent on moderate-to-vigorous physical activity and body image concerns on EA. Regarding the first aim of the study, we found that the large majority of subjects was symptomatic for EA (82%), whereas 11.3% of participants, especially males, were at risk of EA. Although the concept of EA is still not completely described in the literary landscape, some initial studies highlight its incidence among different types of sporty people, such as runners (26%) and team exercisers (15.2%) [15,43], and among other sport disciplines [44,45]. In line with the current findings, Berengüí and colleagues [21], in a sample of Spanish amateur participants, found that, during the COVID-19 pandemic, 6% of the participants showed risk of addiction whereas 74.2% presented some symptoms that may lead to addiction. Furthermore, De la Vega and colleagues [15], in a sample of adults from different countries (Argentina, Chile, Costa Rica, Ecuador, Honduras, Mexico, Spain, and Uruguay), found that, during the pandemic, 15.2% of the subjects were at risk of addiction and 79.6% were symptomatic of EA. In a study conducted on a sample of Italian sporty adults during the first phase of the pandemic (April–May 2020), Ceci and colleagues [46] found that the rate of EA was 4.1%. To our knowledge, no previous data about the prevalence of EA during the second wave of the COVID-19 pandemic among Italian sporty people has been published yet.

Regarding the second aim of the study, when we examined the time spent on moderate and vigorous physical activity, sociocultural attitudes toward appearance and DL as predictors of EA in the regression model, we found that only vigorous activity was linked to EA. When we added drive for leanness and attitudes towards appearance, they significantly added to the vigorous activity in predicting EA. Taken together, these findings suggest that the body image variables add an important element beyond the physical activity level to understand the characteristics of EA during the pandemic, consistent with some research on EA in the pre-pandemic period [26,47].

Although preliminary, these findings may indicate that the desire to achieve a thin, ideal, and perfect body might make vulnerable individuals not only to be inclined to spend more time exercising, but also at risk of compulsive exercise. These results are in line with the prior research conducted in the pre-pandemic period [26,47]. Moreover, the findings of the study may reflect a hierarchical structure for risk, in which individual factors (e.g., drive for leanness and sociocultural attitudes towards appearance) are more relevant than behavioral factors (i.e., time spent on moderate-to-vigorous physical activity) to explain the characteristics of EA.

Prior research showed that COVID-19-related pressures and stress decreased coping resources to control threats to body image and may have increased the exhibition of thin and athletic ideals through social network sites, the only useful tools for maintaining relationships during the pandemic [8,25]. Indeed, social media often encourages exercise as a means of achieving both the thinness and the perfection that create the current body ideal. For example, Instagram explicitly endorses exercise to lose weight and sculpt body parts with pages dedicated to weight loss and exercising for hours daily to achieve radical weight loss [48]. The media strongly suggests that the thin, firm ideal is attained for anyone prepared to spend the time and energy to ‘‘work out’’. Such a phenomenon has been increasingly linked to internalization of appearance ideals and other psychological features linked to compulsive exercise, resulting in the need to explore this trend in gyms during the COVID-19 pandemic, because little research on the subject has been conducted [21]. The results of this study also suggest that the focal point of sociocultural attitudes towards appearance should focus on the potentially more damaging thin ideal.

Study limitations when interpreting the results can be summarized as follows: (1) the cross-sectional design of this study limits the possibility of drawing causal conclusions; (2) the assessment of a non-stratified population with different recruitment procedures as well as the use of an exclusive online sampling strategy (due to restrictions resulting from the pandemic) does not make the data generalizable; (3) the self-report assessment of EA may also limit the conclusions obtained from these results; and (4) participants self-reported their weight and height, and a bias in BMI measurements cannot be excluded. Additionally, BMI is not able to provide information related to real body composition and it does not provide any information about how weight actually impacts an individual’s propensity towards the thin and firm ideal. Despite these limitations, the current study has some strengths, such as the focus on underexamined predictors of EA among sporty people and the use of validated questionnaires for measuring EA and body image concerns. Future research needs to focus on longitudinal designs with stratified samples and include objective assessments of exercise behaviors, BMI, and body composition. Finally, although we examined EA among sporty people, we recommend that the future research investigates the link between EA and body image variables among specific vulnerable groups, such as individuals at risk of eating disorder pathology. There is initial evidence that, during the pandemic, individuals with eating disorders experienced a worsening of symptoms, shape concerns, eating concerns, and increased thinking about exercising [49], possibly as an attempt to reduce COVID-19-related anxiety [8].

## 5. Conclusions

This study is the first to explore the relationship between physical activity level, body image variables, and the deleterious outcome of compulsive exercise during the second wave of the pandemic in an Italian sample of sporty people. High levels of EA are a serious problem, which might have a great impact on public health [21]. Further research to examine the effects of COVID-19 on EA is warranted, given the unique and evolving circumstances related to the pandemic. In conclusion, the results obtained from the current study highlight the importance of considering body image factors in the investigation of EA in sporty people. Culturally, interventions and clinical programming regarding the potential concerns of the sociocultural attitudes and thin body ideal are needed for this seemingly high-risk sample.

## Figures and Tables

**Table 1:** Participants’ demographics and health-related data.

	Participants (N = 194)
Age, M (SD)	25.91 (6.32)
Gender, N (%)	
Females	94 (48.5)
Males	100 (51.5)
Level of education, N (%)	
13 years of education	87 (44.8)
Degree or post-degree	106 (54.6)
Missing	1 (0.5)
Marital status, N (%)	
Married/in a relationship	94 (48.5)
Single	96 (49.5)
Missing	4 (2.0)
BMI, M (SD)	22.23 (3.87)

Note: BMI = body mass index.

**Table 2:** Means, standard deviation, skewness, kurtosis, and correlations.

	M	SD	Skewness	Kurtosis	1	2	3	4
1—Exercise addiction (EAI)	18.49	3.99	0.136	−0.274	-			
2—Sociocultural attitudes towards appearance (SATAQ4)	30.03	8.32	−0.207	−0.423	0.292 **	-		
3—Drive for leanness (DLS)	23.37	6.31	−0.165	−0.548	0.312 **	0.583 **	-	
4—Time spent in vigorous activity (GPAQ)	42.98	48.66	0.164	−1.494	0.195 **	0.008	−0.007	-
5—Time spent on moderate activity (GPAQ)	54.79	47.16	0.993	2.350	0.159 *	0.052	0.107	0.256 **

Note: EAI = Exercise Addiction Inventory; SATAQ4 = Sociocultural Attitudes Towards Appearance Questionnaire-4; DLS = Drive for Leanness Scale; GPAQ = Global Physical Activity Questionnaire = minutes in a typical week. * *p* < 0.05, ** *p* < 0.01.

**Table 3:** Hierarchical regression analyses.

Participants (N = 194)						
	Adj R^2^	ΔR^2^	F	ΔF	β	95% CI
Step 1	−0.008	0.003	0.255	0.255		
Age					0.017	−0.080–0.102
Gender					−0.048	−1.541–0.765
Step 2	0.031	0.049	2.529 *	4.793 **		
Age					−0.013	−0.098–0.082
Gender					−0.015	−1.279–1.045
Time spent on vigorous activity (GPAQ)					0.163 *	0.010–0.280
Time spent on moderate activity (GPAQ)					0.120	−0.002–0.023
Step 3	0.139	0.114	6.092 ***	12.588 ***		
Age					−0.029	−0.104–0.067
Gender					−0.006	−1.057–1.147
Time spent on vigorous activity (GPAQ)					0.179 *	0.032–0.287
Time spent on moderate activity (GPAQ)					0.087	−0.004–0.019
Sociocultural attitudes toward appearance (SATAQ4)					0.166 *	0.000–0.161
Drive for leanness (DLS)					0.215 *	0.030–0.245

Note: GPAQ = Global Physical Activity Questionnaire; SATAQ4 = Sociocultural Attitudes Towards Appearance Questionnaire-4; DLS = Drive for Leanness Scale * *p* < 0.05; ** *p* < 0.01; *** *p* < 0.001.

## Data Availability

Data available on request from the authors.
